# Faecal Microbiota Are Related to Insulin Sensitivity and Secretion in Overweight or Obese Adults

**DOI:** 10.3390/jcm8040452

**Published:** 2019-04-04

**Authors:** Negar Naderpoor, Aya Mousa, Luisa F. Gomez-Arango, Helen L. Barrett, Marloes Dekker Nitert, Barbora de Courten

**Affiliations:** 1 Monash Centre for Health Research and Implementation, School of Public Health and Preventive Medicine, Monash University, Diabetes and Vascular Medicine Unit, Monash Health, Clayton 3168, Australia; negar.naderpoor@monash.edu; 2 Monash Centre for Health Research and Implementation, School of Public Health and Preventive Medicine, Monash University, Clayton 3168, Australia; aya.mousa@monash.edu; 3 School of Chemistry and Molecular Biosciences, University of Queensland, St Lucia 4072, Australia; lulag177@hotmail.com (L.F.G.-A.); m.dekker@uq.edu.au (M.D.N.); 4 Endocrinology department and Mater Research, Mater Hospital, South Brisbane 4101, Australia; Helen.barrett@mater.uq.edu.au

**Keywords:** faecal microbiota, body mass index, percent body fat, insulin secretion, insulin sensitivity, hyperinsulinaemic-euglycaemic clamp

## Abstract

Emerging evidence suggests a role for the gut microbiota in glucose metabolism and diabetes. Few studies have examined the associations between the faecal microbiome and insulin sensitivity and secretion using gold-standard methods in high-risk populations prior to diabetes onset. We investigated the relationships between faecal microbiota composition (16S rRNA sequencing) and gold-standard measures of insulin sensitivity (hyperinsulinaemic-euglycaemic clamp) and insulin secretion (intravenous glucose tolerance test) in 38 overweight or obese otherwise healthy individuals. Genus *Clostridium* was positively associated with insulin sensitivity, and genera *Dialister* and *Phascolarctobacterium* were related to both insulin sensitivity and secretion. Insulin sensitivity was associated with a higher abundance of *Phascolarctobacterium* and lower abundance of *Dialister*. Those with higher insulin secretion had a higher abundance of *Dialister* and lower abundance of *Bifidobacterium*, compared to those with lower insulin secretion. Body mass index (BMI) was positively correlated with *Streptococcus* abundance whereas *Coprococcus* abundance was negatively correlated to BMI and percent body fat. These results suggest that faecal microbiota is related to insulin sensitivity and secretion in overweight or obese adults. These correlations are distinct although partially overlapping, suggesting different pathophysiological pathways. Our findings can inform future trials aiming to manipulate gut microbiome to improve insulin sensitivity and secretion and prevent type 2 diabetes.

## 1. Introduction

The worldwide dramatic rise in the prevalence of both obesity [1] and diabetes [2] calls for further studies to improve our understanding of mechanisms involved in the development of these conditions and to discover therapeutic approaches to reduce the related health and economic burdens. Insulin resistance is a key link between obesity and type 2 diabetes (T2DM) and contributes to increased cardiometabolic risk [3]. Pancreatic β-cell dysfunction, where β-cells are unable to compensate for the degree of insulin resistance by over-secreting insulin, is another key factor in the pathophysiology of T2DM [4]. Recent evidence indicates a role for the gut microbiota in energy homeostasis and insulin resistance [5,6]. Significant differences in faecal microbiota have been demonstrated between individuals with normal and impaired glucose tolerance [7] as well as between those with and without diabetes [8]. Importantly, modifying the gut microbiota using probiotic supplementation or faecal transplant has been shown to alter insulin sensitivity and secretion in both animal and human studies [9,10,11], indicating that the gut microbiota may drive some of the physiological processes associated with altered insulin sensitivity and secretion. It is noteworthy that most studies have focused on either insulin sensitivity or secretion and used related surrogate markers instead of direct measurements.

Findings mainly from animal and in vitro studies have established some of the underlying mechanisms by which the gut microbiota can alter insulin resistance and risk of diabetes [5,6,12]. Lipopolysaccharides (LPS) from Gram-negative bacteria can stimulate the innate immune system by activating toll-like receptors (TLRs) and inducing the release of inflammatory cytokines. LPS also promotes the activation of the nuclear factor *kappa*-B (NFkB) and c-Jun N-terminal kinase (JNK) pathways, both of which have been linked to the development of insulin resistance and impaired insulin signalling in muscle, adipose tissue, liver, and hypothalamus [13]. Importantly, reducing metabolic endotoxaemia, defined by elevated plasma LPS levels, with the use of antibiotics has been shown to improve glucose metabolism, adipose tissue inflammation, and weight gain in high-fat-fed mice [14]. On the other hand, the gut microbiota is involved in the production of short chain fatty acids (SCFAs) including butyrate, propionate, and acetate [15] by fermenting mainly indigestible carbohydrates in the colon. SCFAs, particularly butyrate, have been shown to improve insulin sensitivity and secretion [16] by stimulating glucagon-like peptide 1 (GLP-1) secretion [17] and reducing adipocyte inflammation [18]. Additionally, gut microbiota is important in bile acid metabolism and can produce secondary bile acids, which may act as signalling molecules to modulate signalling through nuclear receptor farnesoid X receptor (FXR) and the G-protein-coupled receptor TGR5 [19]. In mice, the activation of FXR results in impaired glucose homeostasis [20], whereas TGR5 activation improves glucose tolerance by increasing GLP-1 [21] and promoting energy expenditure in brown adipose tissue and muscle [22].

However, most of the current literature on microbiota and insulin resistance or obesity comes from animal studies. In humans, studies are confounded by co-morbidities or the concurrent use of medications and often lack gold-standard methodology for assessing adiposity, insulin resistance and secretion. Therefore, we examined differences in microbiota composition in a cohort of non-diabetic overweight or obese adults in whom obesity, insulin sensitivity and insulin secretion were characterised using dual X-ray absorptiometry, hyperinsulinaemic-euglycaemic clamps and intravenous glucose tolerance tests, respectively.

## 2. Experimental Section

This study uses baseline data from 38 non-diabetic overweight or obese participants who took part in a previous randomised controlled trial (RCT), for which the primary outcomes have been published [23]. Using these baseline data, we investigated correlations between faecal microbiota composition and insulin sensitivity and secretion. Volunteers were recruited from the community in Melbourne through advertisement. Inclusion criteria were the following: age 18 to 60 years, body mass index (BMI) ≥ 25 kg/m^2^, vitamin D deficiency (25-hydroxyvitamin D ≤ 50 nmol/L), stable weight for at least 12 months (weight change <5 kg) with no intention to change the diet or physical activity or to lose weight for the study duration, and with no known or newly diagnosed co-morbidities based on medical or laboratory exam. Exclusion criteria included smoking; weekly alcohol consumption >4 standard drinks for males and >2 standard drinks for females; diabetes based on oral glucose tolerance test (OGTT); use of any medications or supplements; pregnant, lactating, or peri-menopausal women; and/or the presence of acute inflammation based on clinical history or blood test. Ethics approval was obtained from Monash Health and Monash University Human Research Ethics Committees, Melbourne, Australia (Protocol ID: CF13/3874-2013001988). All participants provided written informed consent.

Each participant underwent a physical examination, 75 g OGTT to exclude diabetes based on WHO criteria [24], and a blood test after a 12-hour overnight fast to check full blood count and liver and renal function to exclude other co-morbidities. Validated questionnaires were used to collect data on physical activity (International Physical Activity Questionnaire [25]) and food intake (3-day food diary; Foodworks 8.0 Professional; Xyris Software). 

### 2.1. Anthropometric Measurements

Body mass index was calculated as weight (kg) divided by height squared (m^2^). Dual-energy X-ray absorptiometry (DXA) (Lunar Radiation Corp., Madison, WI, USA) was used to determine body composition.

### 2.2. Insulin Sensitivity and Secretion

Intravenous glucose tolerance tests (IVGTTs) were conducted to measure insulin secretion as an index of both β-cell function and insulin resistance [26,27]. Fifty gram of 50% glucose bolus was administered over 3 minutes to trigger an insulin secretory response. Venous blood samples were collected at –10, 0, 3, 4, 5, 6, 8, 10, 15, 20, 25 and 30 minutes after the glucose bolus to check glucose and insulin concentrations and to calculate the area under the curve (AUC) using the trapezoidal method [28].

To quantify insulin sensitivity, we performed hyperinsulinaemic-euglycaemic clamps as per our protocol [29]. In summary, an intravenous Actrapid bolus (9 mU/kg) was injected and followed by a constant insulin infusion at a rate of 40 mU/m^2^/min for a minimum of 120 minutes with concurrent glucose infusion which was adjusted according to venous blood glucose readings every 5 minutes to maintain blood glucose levels at about 5 mmol/L. Insulin sensitivity (M-value) was defined as the weight-adjusted glucose infusion rate during the last 30 minutes of the clamp and when a steady state was achieved. Plasma glucose was measured by the glucose oxidase method (YSI 2300 STAT, YSI Inc., Yellow Springs, OH, USA) and plasma insulin was determined using commercial enzymatic immunoassays (Beckman Coulter, Lane Cove, Australia). Participants were instructed not to consume coffee, tea, chocolate, and alcohol for at least 24 hours prior to the metabolic tests.

### 2.3. Microbiota Analysis

Bacterial DNA was extracted from 0.25 g of thawed stool samples using the repeated bead beating method (RBB+C) followed by the Maxwell 16 Blood DNA purification kit (Promega, Madison, WI, USA). Mechanical lysis was completed using a mixture of 0.1 and 0.5 mm sterile zirconia beads for homogenisation for 3 minutes at 30 Hz in the TissueLyser II (Qiagen, Chadstone Centre, Australia). Purified DNA was quantified by the Nanodrop ND 1000 spectrophotometer (ThermoFisher Scientific, Scoresby, Australia).

The 16S rRNA gene amplicon sequencing was achieved using the Illumina MiSeq platform at the Australian Centre for Ecogenomics at the University of Queensland. The V6-V8 region of the 16S rRNA gene was amplified using the primer set 926F (5′-TCG TCG GCA GCG TCA GAT GTG TAT AAG AGA CAG AAA CTY AAA KGA ATT GRC GG-3′) and 1392R (5′-GTC TCG TGG GCT CGG AGA TGT GTA TAA GAG ACA GAC GGG CGG TGW GTR C-3′). Amplicons were barcoded using the Nextera XT V2 Index Kit Set A and further purified using AMPure XP beads per manufacturer’s instructions. Barcoded amplicons were quantified, normalised and pooled. Sequences were joined, demultiplexed and quality filtered using QIIME (Quantitative Insights Into Microbial Ecology) software suite version 1.9.1 (www.qiime.org). Clustering to operational taxonomic units (OTUs) was achieved using the open reference OTU picking method using the Greengenes database with a 97% pairwise identity threshold. OTUs with an overall abundance <0.0001 were removed from the resultant OTU table. Data were normalised using the cumulative sum scaling (CSS) method.

### 2.4. Statistical Analysis

Statistical analysis for clinical data was performed using IBM SPSS Statistics version 24, Armonk, NY, USA. Data are presented as mean ± SD or median (interquartile range) for normally and non-normally distributed variables, respectively. Histograms and Shapiro-Wilk tests were used to assess normality of variable distributions. We used the M-value of 4.7 mg/kg/min [30] and median insulin AUC of 1516.43 mU/L as cut-offs to categorise our study sample to insulin-resistant and insulin-sensitive groups or to groups with higher or lower insulin secretion.

For microbiota analyses, fasting insulin, insulin AUC, M-value, BMI and percent body fat were correlated with taxa at different taxonomic levels with bootstrapped Spearman rank correlation tests. Differences in microbiota composition based on insulin sensitivity and secretion categories were evaluated by Kruskal Wallis tests. Microbial diversity within groups (α-diversity) was measured by the Shannon Index and microbial diversity between groups (β-diversity) was determined by CCA (Canonical Correspondence Analysis). Results were not adjusted for multiple testing.

## 3. Results

### 3.1. Baseline Characteristics

Thirty-eight participants including 22 males and 16 females were included in the analysis. Baseline characteristics are presented in Table 1. Participants were aged between 18 to 57 years (mean 33.13 ± 9.09 years) from ethnically diverse backgrounds. Two participants did not have IVGTTs and one participant did not have a measurement of insulin sensitivity by the clamp. Impaired fasting glucose and impaired glucose tolerance were diagnosed in one and four participants, respectively.

There was a negative relationship between insulin sensitivity (M-value) and fasting insulin (*r* = −0.6, *p* < 0.001) and insulin AUC (*r* = −0.46, *p* = 0.005) but not with fasting or 2-hour post OGTT blood glucose levels.

**Figure 1:**
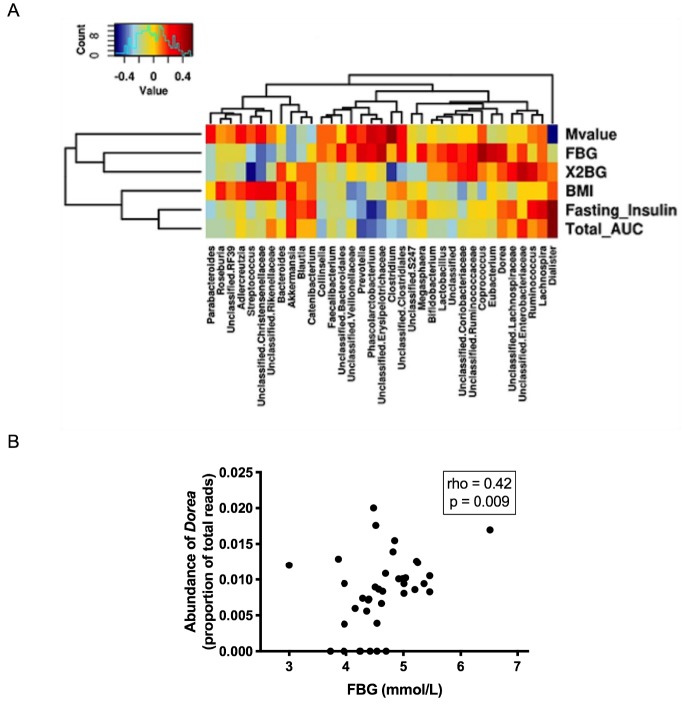
(**A**) The heat map of the Spearman correlations between faecal microbiota at the genus level and insulin sensitivity/secretion, glycaemic parameters, and BMI. (**B**) The relationship between fasting blood glucose and genus *Dorea*. BMI: body mass index, FBG: fasting blood glucose level, x2BG: 2-hour blood glucose level post-oral glucose tolerance test, Total AUC: total insulin area under the curve.

Figure 1A illustrates a heat map of the associations of different genera with fasting insulin, total insulin AUC, and M-value as well as BMI, and fasting and 2-hour post-OGTT blood glucose levels. The colour scale from blue to red indicates the lowest (negative) correlation to the highest (positive) correlation between the above-mentioned variables and various genera. For example, fasting blood glucose was positively correlated with the abundance of the genus *Dorea* and this relationship is also shown in Figure 1B (rho = 0.42, *p* = 0.009).

### 3.2. Association between Faecal Microbiota and Insulin Sensitivity

There were no significant differences in diet composition (total energy intake, carbohydrate, protein, fat, saturated fat, and fibre intake), or in physical activity between insulin-sensitive and insulin-resistant groups (all *p* > 0.1).

**Figure 2:**
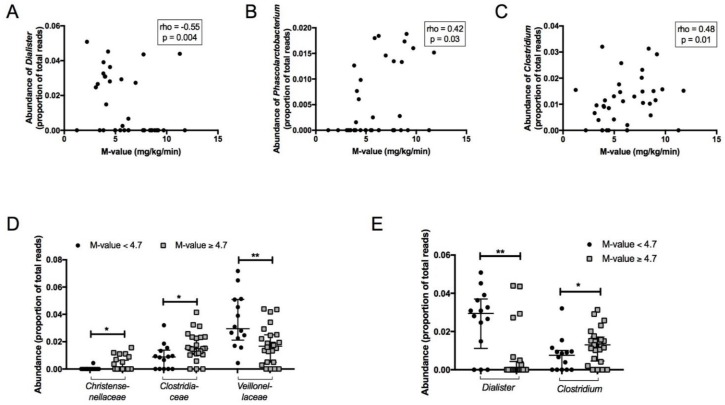
(**A**) The Spearman correlation between insulin sensitivity (M-value) and *Dialister*. (**B**) Spearman correlation between insulin sensitivity (M-value) and *Phascolarctobacterium.* (**C**) Spearman correlation between insulin sensitivity (M-value) and *Clostridium*. (**D,E**) Differences in faecal microbiota between insulin resistant (M < 4.7 mg/kg/min) and insulin sensitive (M ≥ 4.7 mg/kg/min) groups at family (**D**) and genus (**E**) levels.

Abundance of the genus *Dialister* was negatively correlated (rho = −0.55, *p* = 0.004) while genera *Clostridium* (rho = 0.48, *p* = 0.01) and *Phascolarctobacterium* (rho = 0.42, *p* = 0.03) were positively correlated with M-value (Figure 2A–C). There was no significant correlation between *Akkermansia muciniphila* and M-value (*p* = 0.16).

No significant differences in α-diversity were found between insulin-resistant (M-value < 4.7 mg/kg/min, *n* = 14) and insulin-sensitive (M-value ≥ 4.7 mg/kg/min, *n* = 23) individuals. Differences between these two groups at the family and genus taxonomic levels are presented in Figure 2D,E. At the family level, the insulin-resistant group had a significantly higher abundance of *Veillonellaceae* and lower abundance of *Christensenellaceae* and *Clostridiaceae* compared with the insulin-sensitive group (Figure 2D). At the genus level, *Dialister* was more abundant and *Clostridium* was less abundant in the insulin-resistant group (Figure 2E).

### 3.3. Association between Faecal Microbiota and Insulin Secretion

There were no significant differences in diet composition or physical activity between groups with high and low insulin AUC (all *p* > 0.09).

**Figure 3:**
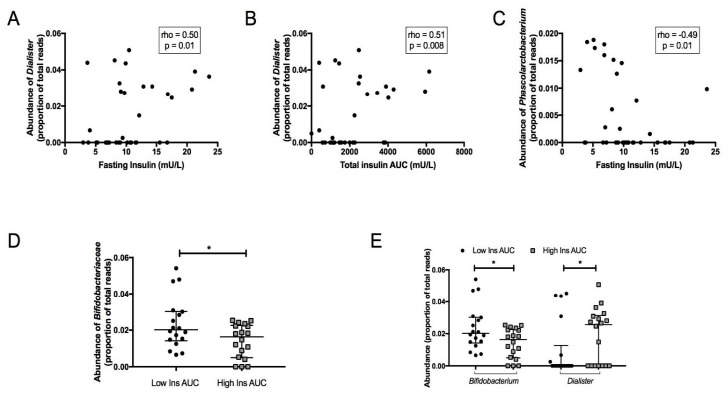
(**A**) The Spearman correlation between fasting insulin and *Dialister*. (**B**) Spearman correlation between total insulin AUC and *Dialister*. (**C**) Spearman correlation between fasting insulin and *Phascolarctobacterium*. (**D,E**) Differences in faecal microbiota between higher and lower insulin secretion groups at family (**D**) and genus (**E**) levels. Boxes represent the interquartile ranges for relative abundances, lines inside the boxes show medians. * *p*-value < 0.05, ** *p*-value < 0.01.

Abundance of the genus *Dialister* was positively correlated with fasting insulin (rho = 0.50, *p* = 0.01) (Figure 3A) and insulin AUC (rho = 0.51, *p* = 0.008) (Figure 3B), whereas abundance of the genus *Phascolarctobacterium* was negatively correlated with fasting insulin (rho = −0.49, *p* = 0.01) (Figure 3C). At the family level, *Bifidobacteriaceae* were more abundant in individuals with higher insulin AUC (Figure 3D). At the genus level, *Dialister* had a higher abundance in the group with higher insulin AUC and *Bifidobacterium* was more abundant in the group with lower insulin AUC (Figure 3E).

### 3.4. Association between Faecal Microbiota and Anthropometric Parameters

**Figure 4:**
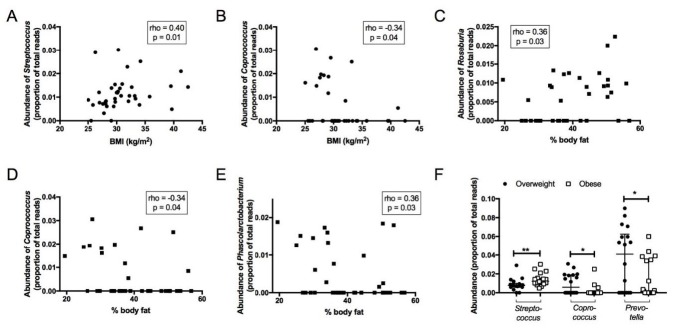
(**A**) The Spearman correlation between BMI and *Streptococcus.* (**B**) Spearman correlation between BMI and *Coprococcus.* (**C**) Spearman correlation between %body fat and *Roseburia.* (**D**) Spearman correlation between %body fat and *Coprococcus.* (**E**) Spearman correlation between %body fat and *Phascolarctobacterium.* (**F**) Differences in faecal microbiota between overweight and obese groups.

BMI was positively correlated with the abundance of *Streptococcus* (rho = 0.39, *p* = 0.02) (Figure 4A) and negatively correlated with *Coprococcus* abundance (rho = −0.35, *p* = 0.03) (Figure 4B). Furthermore, percent body fat was positively correlated with the abundance of the SCFA producer *Roseburia* (rho = 0.36, *p* = 0.03) (Figure 4C) and negatively with *Phascolarctobacterium* (rho = 0.36, *p* = 0.03) (Figure 4D) and *Coprococcus* (rho = −0.34, *p* = 0.04) (Figure 4E). Comparing the faecal microbiota in overweight (BMI: 25–29.9 kg/m^2^, *n* = 18) versus obese (BMI ≥ 30 kg/m^2^, *n* = 20) individuals, there were no significant differences in α- and β-diversity. However, the obese group had a higher abundance of genus *Streptococcus* and lower abundance of genus *Coprococcus* and *Prevotella* compared with the overweight group (Figure 4F).

## 4. Discussion

In this study, we report associations between faecal microbiota composition and gold-standard measures of adiposity, insulin sensitivity and secretion in a metabolically well-characterized, community-based sample of overweight or obese otherwise healthy individuals. Differences in faecal microbial communities at the family and genus taxonomic levels were observed between insulin-sensitive and insulin-resistant groups and between those with higher and lower insulin secretion. Some of these associations overlap while others are specific to insulin sensitivity or secretion, suggesting that different mechanisms might be involved. To the best of our knowledge, this is the first study examining the correlations of faecal microbiota with both insulin sensitivity and insulin secretion in individuals at high risk for diabetes.

The abundance of *Dialister* was negatively correlated with insulin sensitivity (measured as M-value by hyperinsulinaemic-euglycaemic clamp) as well as positively with insulin concentrations both in the fasting and glucose-stimulated states (measured as total insulin AUC by IVGTT). In addition, *Dialister* abundance was increased in those participants with a total insulin AUC above the median and in those with M-values below 4.7 mg/kg/min. The overlap between these categories was large, whereby 61% (11/18) of individuals with a high total insulin AUC had an M-value < 4.7 mg/kg/min. *Dialister* abundance has been previously associated with abnormal glucose metabolism. A higher abundance of genus *Dialister* has been reported among overweight or obese African-American men with impaired glucose tolerance (IGT) compared to those with normal glucose tolerance (NGT) [7]. Its abundance was also higher in patients with coronary artery disease who were diabetic compared to non-diabetic [31]. Furthermore, in a small cohort of patients with type 2 diabetes who underwent bariatric surgery, the abundance of *Dialister* was reduced [32]. Dialister is a member of the Veillonellaceae family, which showed a decreased abundance in insulin-sensitive individuals in our cohort. However, *Dialister* abundance was not different in overweight versus obese individuals indicating that *Dialister* abundance may be more reflective of insulin sensitivity rather than BMI. *Dialister* is a Gram-negative coccobacillus, which has been shown to be capable of producing acetate, lactate and propionate but not butyrate [33]. Therefore, *Dialister* could potentially have both negative (inflammatory) and positive (via SCFA) effects on the host and the overall effect may be determined through interactions with other bacteria.

In our study population of overweight or obese adults, participants with lower insulin secretion had a higher abundance of genus *Bifidobacterium*. *Bifidobacterium* has been reported in animal and human studies to have beneficial effects in metabolic syndrome and inflammation by improving the mucosal barrier function in the gut, thereby reducing intestinal endotoxin levels as well as endotoxaemia [34,35]. *Bifidobacterium* is shown to be lower in abundance in patients with diabetes compared to healthy individuals, in insulin-resistant versus insulin-sensitive morbidly obese individuals, and in pregnant women with gestational diabetes compared to healthy pregnant women [36,37,38]. Restoration of *Bifidobacterium spp*. using prebiotics reduced insulin concentration and improved glucose tolerance in mice and overweight humans [35,39]. Notably, *Bifidobacterium* was not more abundant in the insulin-sensitive compared with insulin-resistant groups in our cohort. Our findings suggest that *Bifidobacterium* may have a stronger link to insulin secretion rather than insulin sensitivity; however, confirmation of this relationship awaits further study.

In our study, genus *Phascolarctobacterium* (from family *Veillonellaceae* and order *Clostridiales)* was positively related to insulin sensitivity, and negatively related to fasting insulin. *Phascolarctobacterium* is an SCFA producer and previous animal and human studies investigating relationships with markers of insulin sensitivity have reported inconsistent results, with some showing negative [38,40] and others showing positive correlations [36,41]. Interestingly, in our study, the correlations between *Phascolarctobacterium* and insulin sensitivity, and fasting insulin consistently pointed toward a positive relationship. Our results are consistent with a study in morbidly obese individuals with varying levels of insulin sensitivity which demonstrated a higher abundance of *Phascolarctobacterium* in appendix samples of insulin-sensitive compared to the insulin-resistant group [36]. In addition, we found a negative correlation between *Phascolarctobacterium* and percent body fat; excess fat mass is associated with decreased insulin sensitivity and the negative association of body fat percentage with *Phascolarctobacterium* likely reflects this relationship with insulin resistance. On the contrary, another human study reported a higher abundance of *Phascolarctobacterium* in women with gestational diabetes; however, due to hormonal and metabolic changes of pregnancy, these results may not be generalizable to non-pregnant individuals.

In addition, we found no correlation between *Akkermansia muciniphila* and insulin sensitivity. *Akkermansia muciniphila* is a butyrate-producing bacteria from the phylum *Verrucomicrobia* which has been shown in some studies to be positively related to insulin sensitivity and negatively related to BMI [1]. This may be due to the small sample size and/or including only overweight or obese individuals in our study.

In this cohort, the abundance of *Streptococcus* was positively correlated with BMI and increased in obese individuals. This has been reported previously in a cohort of school-aged children [42] and in patients with type 2 diabetes suffering from coronary heart disease [31]. *Streptococcus* is localised to the gut mucosa rather than the lumen, possibly due to their tolerance to oxygen and ability to decrease PH locally, thereby contributing to protection from pathological microorganisms [43]. *Streptococcus* abundance has been reported to be negatively correlated to HDL-cholesterol levels in the circulation which indicates that these bacteria may act on the lipid metabolism in the host [31]. At the same time, *Coprococcus* abundance showed the reverse trend with a negative correlation with BMI and percent body fat and increased abundance in overweight compared to obese individuals. Other studies have also reported a lower abundance of *Coprococcus* in normal-weight versus obese individuals [44,45]. Lastly, *Prevotella* abundance was lower in obese participants in our cohort. Increasing *Prevotella* abundance has been associated with improved glucose tolerance in normal-weight and overweight individuals who responded to a dietary intervention with barley kernels [46]. This suggests that higher *Prevotella* abundance may have beneficial effects on the metabolic health of the host.

Our study has some limitations. The observational design does not allow for causal inference. In addition, the correlations between faecal microbiota and insulin sensitivity and secretion were not the primary outcomes of this study and therefore, the sample size was not determined to examine these correlations. Due to the small sample size, we were not able to adjust for multiple testing or ethnicity. Furthermore, data on stool consistency was not available for our participants and this might have had an impact on faecal microbiota composition and richness [47]. Additionally, we cannot eliminate the impact of vitamin D deficiency on the faecal microbiota in this cohort. However, this is unlikely to have affected our results or differences between groups since all participants were vitamin D-deficient. Finally, functional analysis of the faecal microbiota and measurements of incretins or other biomarkers were not performed in this study which could have shed more light on the possible mechanisms underlying the correlations found between faecal microbiota and insulin sensitivity and secretion.

Nevertheless, our study findings provide further evidence to support some of the previously reported associations of faecal microbiota with insulin sensitivity and add to the current literature by showing some new associations based on the gold-standard methodology for assessment of insulin sensitivity and secretion. We demonstrate that associations between faecal microbiota and insulin sensitivity based on M-value measured by hyperinsulinaemic-euglycaemic clamp partially overlap and partially differ from associations with insulin secretion measured by IVGTT. This suggests that discrete mechanisms may underlie the pathophysiology of insulin resistance and β-cell dysfunction and progression to diabetes. As such, preventive modulation of the gut microbiota may need to target different bacteria in those with IGT compared with impaired fasting glucose as insulin resistance has been shown to be more predictive of IGT while impaired insulin secretion is more predictive of IFG [48]. This hypothesis needs to be investigated by interventional studies.

## Figures and Tables

**Table 1:** The participants’ characteristics.

Variable	N (%)
Ethnicity (self-reported)	
Caucasian	8 (21.1%)
South and Central Asia	13 (34.2%
Southeast and Northeast Asia	11 (29.0%)
Others *	5 (13.2%)
Missing	1 (2.6%)
	Mean ± SD or Median (IQR)
Age (years)	33.13 ± 9.09
BMI (kg/m^2^)	30.07 (4.70)
Percent body fat	40.53 ± 9.24
M-value (mg/kg/min)	6.07 ± 2.52
Insulin AUC (mU/L)	1516.43 (1714.38)
Fasting insulin (mU/L)	9.55 (5.74)
Fasting BGL (mmol/L)	4.63 ± 0.61
2-hour BGL post OGTT (mmol/L)	5.35 (1.95)
Total daily energy intake (kJ)	7584.00 (2845.33)
Daily carbohydrate intake (g)	222.93 ± 72.06
Daily protein intake (g)	88.30 (42.55)
Daily fat intake (g)	67.01 (41.05)
Daily saturated fat intake (g)	22.61 (13.67)
Daily fibre intake (g)	17.27 (10.03)
IPAQMETs	2856.00 (3771.00)

* Others: African, Middle Eastern, South American, Polynesian. Median [interquartile range] for not normally distributed variables. BMI: body mass index, AUC: area under the curve, BGL: blood glucose level, IQR: interquartile range, OGTT: oral glucose tolerance test, IPAQMETs: international physical activity questionnaire multiples of the metabolic rate.
